# A Quantitative Analysis of *Micrurus* Coral Snakes Reveals Unexpected Variation in Stereotyped Anti-Predator Displays Within a Mimicry System

**DOI:** 10.1093/iob/obaa006

**Published:** 2020-03-02

**Authors:** T Y Moore, S M Danforth, J G Larson, A R Davis Rabosky

**Affiliations:** 1 Robotics Institute, University of Michigan, 2350 Hayward St, Ann Arbor, MI 48109, USA; 2 Ecology and Evolutionary Biology, University of Michigan, 1105 N. University Ave, Ann Arbor, MI 48109, USA; 3 Museum of Zoology, University of Michigan, 3600 Varsity Drive, Ann Arbor, MI 48108, USA; 4 Mechanical Engineering, University of Michigan, 2350 Hayward St, Ann Arbor, MI 48109, USA

## Abstract

Warning signals in chemically defended organisms are critical components of predator–prey interactions, often requiring multiple coordinated display components for effective communication. When threatened by a predator, venomous coral snakes (genus *Micrurus*) display a vigorous, non-locomotory thrashing behavior that has previously been qualitatively described. Given the high contrast and colorful banding patterns of these snakes, this thrashing display is hypothesized to be a key component of a complex aposematic signal under strong stabilizing selection across species in a mimicry system. By experimentally testing snake response across simulated predator cues, we analyzed variation in the presence and expression of a thrashing display across five species of South American coral snakes. Although the major features of the thrash display were conserved across species, we found that predator cue type, snake body size, and species identity predict significant inter- and intraspecific variation in the propensity to perform a display, the duration of thrashing, and the curvature of snake bodies. We also found an interaction between curve magnitude and body location that clearly shows which parts of the display vary most across individuals and species. Our results suggest that contrary to the assumption that all *Micrurus* species and individuals perform the same display, a high degree of variation exists despite presumably strong selection to conserve a common signal. This quantitative behavioral characterization presents a new framework for analyzing the non-locomotory motions displayed by snakes in a broader ecological context, especially for signaling systems with complex interaction across multiple modalities.

## Synopsis

Un análisis cuantitativo de las serpientes coral (Genero: *Micrurus*) revela una variación inesperada de los comportamientos anti-depredadores estereotipados dentro de un sistema de mímicoLas señales de advertencia en organismos con defensas químicas son componentes críticos de las interacciones depredador-presa, que a menudo requieren múltiples componentes de visualización coordinados para una comunicación efectiva. Cuando son amenazadas por un depredador, las serpientes de coral venenosas (género *Micrurus*) muestran un comportamiento vigoroso de coletazos, no locomotor, que solo se ha descrito cualitativamente. Dado el alto contraste y los patrones de bandas coloridas de estas serpientes, se supone que este comportamiento de coletazos es un componente clave de una señal aposemática compleja bajo una fuerte selección estabilizadora entre especies en un sistema de mímica. Al probar experimentalmente la respuesta de las serpientes a través de señales simuladas de depredadores, analizamos la variación en la presencia y la expresión de coletazos en cinco especies de serpientes de coral de América del Sur. Aunque las principales características de la muestra se conservaron en todas las especies, encontramos que el tipo de señal de depredación, el tamaño del cuerpo de la serpiente y la especie en si predicen una variación significativa inter e intraespecífica en la probabilidad a realizar la conducta, la duración del coletazo y la curvatura del cuerpo de las serpientes. También encontramos una interacción entre la magnitud de la curva y la ubicación del cuerpo que muestra claramente qué partes del cuerpo varían más entre individuos y especies. Nuestros resultados sugieren que, contrariamente a la suposición de que todas las especies e individuos realizan el mismo comportamiento, persiste un alto grado de variación en el comportamiento de coletazos exhibido por las serpientes de coral *Micrurus* a pesar de una selección presumiblemente fuerte para converger en una señal común. Esta caracterización cuantitativa del comportamiento presenta un nuevo marco para analizar los movimientos no locomotores mostrados por las serpientes en un contexto ecológico más amplio, especialmente para los sistemas de señalización con interacción compleja a través de múltiples modalidades. Translated to Spanish by YE Jimenez (yordano_jimenez@brown.edu)

## Introduction

Venomous prey animals often use conspicuous phenotypes to communicate their lethal toxicity to potential predators (Ruxton et al. 2018). These aposematic signals can be visual, chemical, acoustic, or can involve complex interactions between multiple distinct components, such as color patterning and body motion (Rowe and Halpin 2013; Dalziell and Welbergen 2016). According to the theory of mimicry, toxic prey animals can reinforce the aposematic signal to their predators by converging on a common phenotype, even across multimodal components (e.g., conspicuous color and behavioral display; Wallace 1867; Müller 1878; Sherratt 2008).

**Fig. 1:**
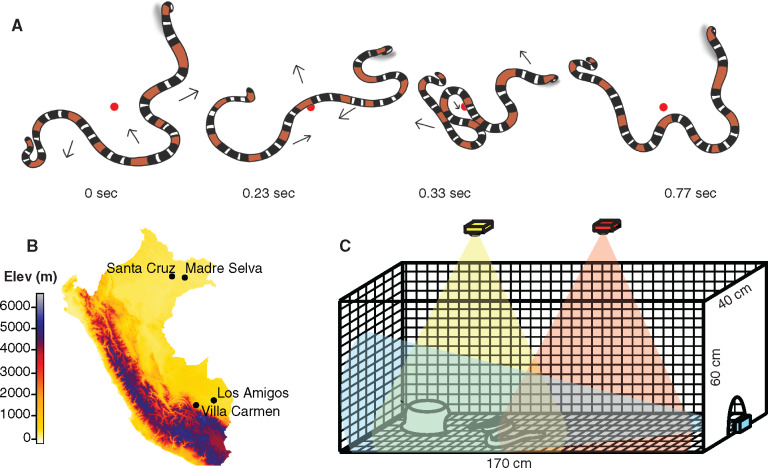
(**a**) An illustration of a coral snake thrashing display. Each of the four illustrations depicts an individual coral snake progressing through different phases of a thrashing display (based on data in this study). Arrows represent direction of movement for corresponding body segments. The snake position with respect to the red dot shows that there is negligible translocation of the snake, despite vigorous movements. The snake head is on the left side of each illustration, and the coiled tail is elevated throughout the display. See Supplementary Video S1 to see a full thrashing trial. (**B**) A map of Peru showing our four field sites, all in lowland Amazonian rainforest. (**C**) A schematic of the pop-up behavioral arena. The bottom and sides of the arena are constructed from corrugated plastic and connected using brass fasteners. Lines are drawn 2 cm apart using permanent ink and covered with a clear adhesive plastic (Con-Tact). Three Go-Pro cameras were positioned so that their fields of view partially overlapped and included the entire floor-space of the arena (shown in yellow, red, and blue). An opaque plastic bowl with a portion removed was placed in one end of the arena to provide a refuge for the snake.

Coral snakes are highly venomous elapid snakes in the genus *Micrurus* that have been described as a mimicry system using visual warning signals of their chemical defense (Campbell and Lamar 2004). Most coral snake species are found in the Neotropics, with the highest sympatric species richness in the Western Amazon Basin (Davis Rabosky et al. 2016a). Coral snakes are well known for their conspicuous red and black coloration (Fig. 1A), a high-contrast banding pattern that effectively creates an aposematic signal deterring avian (Smith 1975; Smith 1977) and potentially mammalian predators (Greene and McDiarmid 1981; Savage and Slowinski 1992; Martins 1996; Buasso et al. 2006). In addition to their bright coloration, coral snakes encountering a threat that also produce a distinctive anti-predator display that includes elements such as body flattening, intermittent thrashing, head hiding, and coiling of the tail, which is often elevated and waved or “waggled” (Greene 1973, 1979; Fig. 1A;Supplementary Video S1). In some species, this display is also accompanied by an auditory cloacal “popping” sound, eversion of the hemipenes, and emission of cloacal musk and feces (Greene 1973; Sazima and Abe 1991). Because such similar elements of this behavioral display have been reported across many coral snake species in both Asia and the Americas (Brown et al. 2013b), this anti-predator response is expected to have (1) an old origin that predates the arrival of this clade in the Western Hemisphere ∼35mya and (2) a significant genetic basis like other homologous traits derived from shared ancestry (Wake et al. 2011). The main purpose of this display has been hypothesized as inducing an effective cognitive illusion that reduces the ability of the attacking predator to identify and target the head (Roze 1996). The intermittent, self-occluding thrashing is described as the foundational display component driving this effect in all species examined.

However, individual-level variation in mimicry traits is also expected to exist, with critical impacts on the ecological and evolutionary dynamics of mimicry over space and time. Variation in the banding elements of coral snake color pattern has been well documented (Davis Rabosky et al. 2016a, 2016b) and experimentally tested using clay replicas for its effect on predator deterrence (Brodie 1993; Buasso et al. 2006; Kikuchi and Pfennig 2010). Critically, both empirical and theoretical studies have shown that model–mimic pairs can experience local, spatially explicit selection pressures that specifically promote either high or low levels of phenotypic (e.g., color pattern) variation, often within a geographic mosaic or clinal pattern (Holmes et al. 2017; Jiggins 2017). Classification of color pattern variation in both mimics and models is often strikingly bimodal, with examples of high intraspecific variation caused through diversifying or negative frequency-dependent selection and examples of low variation caused by strong stabilizing or directional selection (Mallet and Joron 1999). Although the evolutionary persistence of both types of intraspecific variation may seem paradoxical, coral snake mimicry in particular appears to maintain both models and mimics with very low color pattern variation and those with spectacularly high color polymorphism (Davis Rabosky et al. 2016b).

As previous authors have noted, clay replica studies testing the adaptive value of mimicry may not accurately reflect the true deterrence value of a given phenotype because they exclude behavior (Brodie 1993). While forward movement of clay models has reinforced the patterns of prey deterrence in frogs (Paluh et al. 2014), a thrashing display has the potential to greatly alter the perception of a color pattern in snakes (Titcomb et al. 2014; Brodie 1992) and create ambiguity about predicted effects of movement on signal efficacy in this system. Importantly, color pattern and non-locomotory behavior in coral snakes may interact to produce a complex anti-predator signal that is not fully explained by either individual component. This interaction effect might be most pronounced in coral snake species in which color variation is particularly low or high, as species under either strong stabilizing or diversifying selection on color are predicted to have correlated outcomes in behavioral variation when the same selective forces are acting on both traits (e.g., visually oriented avian predators simultaneously assessing color and motion signals; Rowe and Halpin 2013). However, neither the drivers of variation in *Micrurus* anti-predator displays nor the relationship of behavior to color pattern has ever been tested.

While snake locomotion has been quantitatively characterized in a variety of contexts (Lissmann 1950; Jayne 1986; Moon and Gans 1998; Hu et al. 2009; Socha 2011; Titcomb et al. 2014), most studies of non-locomotory snake behaviors rely upon qualitative descriptions (Arnold and Bennett 1984; Brodie 1992). Here we present the first quantitative analysis of non-locomotory anti-predator behaviors in five species of *Micrurus* coral snakes that have low intraspecific variation in color pattern and test for signal-reinforcing similarity within and among species, as predicted by mimicry theory. By characterizing these behaviors in response to experimentally induced predator contexts, we form a functional basis for understanding both the signaling mechanism of the aposematic phenotype and the selective pressures shaping behavioral convergence among species in a mimicry system.

## Methods

### Data collection

All animal-related procedures have been approved by the University of Michigan Institutional Animal Care and Use Committee (Protocols #PRO00006234 and #PRO00008306) and the Peruvian government SERFOR (Servicio Nacional Forestal y de Fauna Silvestre; permit numbers: 029-2016-SERFOR-DGGSPFFS, 405-2016-SERFOR-DGGSPFFS, 116-2017-SERFOR-DGGSPFFS). We collected data during five field expeditions in the Amazonian lowlands of Peru from March 2016 to December 2018, at Villa Carmen, Los Amigos, Madre Selva, and Santa Cruz Biological Stations (Fig. 1B). We captured snakes either in funnel traps or opportunistically during transects, then transported the snakes in fabric bags secured within 20 L lidded buckets back to the station. During capture and handling, all trained personnel were equipped with snake hooks, tongs, venom defender gloves (1-2-1 Products Ltd., Alfreton, UK), and knee-high rubber boots to avoid envenomation.

We recorded anti-predator behavior in a pop-up behavioral arena constructed of corrugated plastic (Fig. 1C) illuminated by a string of LED lights attached to the inner surface at the top edge of the arena walls (see Davis Rabosky et al. 2019, for more details on construction). We marked the inner surface of the arena with visual fiducial markings to aid in the removal of lens distortion and measurement. We covered the inner surface of the arena with an adhesive transparent plastic film (Con-Tact, Rubbermaid, Atlanta, Georgia, USA) to facilitate thorough and rapid cleansing and preserve the visual fiducial markings. Since previous research has shown that snakes are physiologically affected by temperature, chemical cues, and light in an environment (Schieffelin and de Queiroz 1991), it is likely that the behaviors exhibited in laboratory environments and by captive individuals differ significantly from those exhibited under natural conditions. Therefore, we made every effort to ensure similar experimental conditions for all behavioral trials. After capture, snakes were kept undisturbed in bags for <24 h before behavioral trials and the inner surface of the arena was washed with unscented soap and water to limit exposure to the chemical cues of previous experimental subjects.

We immediately began recording the snake behavior as we gently placed snakes into the arena, one at a time, by tipping the cloth bag into the arena without directly handling the snake. After the first trial, we measured surface temperature of each individual with a Raytek Raynger ST81 infrared temperature sensor. We recorded snake behavior using either two or three GoPro (San Mateo, CA) Hero 4+ Black or three Hero 5+ Black cameras filming from overhead and lateral views (see Fig. 1C for camera positions) at 30, 60, or 120 frames per second, depending on the lighting conditions.

We used three different stimuli to elicit anti-predator behaviors. Only one type of stimulus was presented during a given trial, but the stimulus was presented several times throughout the duration of the trial. We recorded snake behaviors for up to 2 min, allowing 1 min of time to rest before a new form of stimulus was presented in the next trial. The order in which these stimuli were presented was randomized for each individual snake. To simulate avian predation threat, we quickly moved a piece of cloth across the top of the arena to create the visual looming and pressure wave stimuli produced by a swooping bird. To simulate a large mammal predator, we used the vibration produced by a cellular phone and placed it in contact with the arena. To test for response after contact with a predator, we used a 1 m snake hook to lightly tap the snake.

After behavioral testing, we either vouchered snakes into the University of Michigan Museum of Zoology (UMMZ) or the Museo de Historia Natural in Lima, Peru (MUSM), or released the snake at the point of capture. All vouchered snakes were also weighed for body mass, measured for snout–vent length (SVL) and tail length, and sexed where possible. Field numbers and museum accession numbers (when available) for each individual are reported in Table 1.

**Table 1:** Morphological and geographical information for the snakes examined in this study

Species	Catalog number	Field number	Sex	SVL (mm)	Mass (g)	Locality	Thrashing?
*Micrurus annellatus*	MUSM 39056	RAB 2815	F	410	17.5	Los Amigos	No
*Micrurus annellatus*	UMMZ 246856	RAB 1144	F	422	12.0	Los Amigos	Yes
*Micrurus annellatus*	UMMZ 248451	RAB 3275	F	497	18.1	Los Amigos	Yes
*Micrurus hemprichii*	UMMZ 246857	RAB 1810	M	740	86.0	Madre Selva	Yes
*Micrurus hemprichii*	MUSM 37347	RAB 2035	M	617	52.5	Madre Selva	Yes
*Micrurus lemniscatus*	Not Applicable	Released5	Not Applicable	Not Applicable	Not Applicable	Villa Carmen	No
*Micrurus lemniscatus*	Not Applicable	Released9	Not Applicable	848*	Not Applicable	Villa Carmen	Yes
*Micrurus lemniscatus*	Not Applicable	Released12	Not Applicable	Not Applicable	Not Applicable	Villa Carmen	Not analyzed (out of frame)
*Micrurus lemniscatus*	UMMZ 246858	RAB 1993	F	632	23.4	Madre Selva	Yes
*Micrurus lemniscatus*	MUSM 37348	RAB 2415	F	372	6.1	Santa Cruz	Yes
*Micrurus lemniscatus*	MUSM 39057	RAB 2706	M	816	68.9	Los Amigos	Not analyzed (snake hook interference)
*Micrurus lemniscatus*	UMMZ 248456	RAB 2915	F	688	34.6	Los Amigos	Yes
*Micrurus lemniscatus*	UMMZ 248457	RAB 3333	M	595	35.0	Los Amigos	Yes
*Micrurus lemniscatus*	UMMZ 248452	RAB 3487	F	632	28.4	Los Amigos	Yes
*Micrurus lemniscatus*	MUSM 39853	RAB 3573	M	570	25.7	Los Amigos	Yes
*Micrurus lemniscatus*	MUSM 39854	RAB 3574	M	590	25.5	Los Amigos	Yes
*Micrurus lemniscatus*	MUSM 39855	RAB 3578	M	453	14.1	Los Amigos	Yes
*Micrurus obscurus*	UMMZ 246859	RAB 0665	F	261	5.2	Villa Carmen	Yes
*Micrurus obscurus*	MUSM 37350	RAB 0698	F	237	5.1	Villa Carmen	Yes
*Micrurus obscurus*	UMMZ 246860	RAB 1054	M	775	81.0	Los Amigos	Yes
*Micrurus obscurus*	UMMZ 248458	RAB 3570	F	766	86.0	Los Amigos	Yes
*Micrurus surinamensis*	UMMZ 246861	RAB 1099	F	657	100.0	Los Amigos	No
*Micrurus surinamensis*	MUSM 37352	RAB 1100	F	391	25.2	Los Amigos	Yes
*Micrurus surinamensis*	MUSM 37353	RAB 1101	M	421	32.5	Los Amigos	Yes
*Micrurus surinamensis*	UMMZ 246862	RAB 1511	F	948	240.0	Madre Selva	No

Note that * indicates that the SVL for *M. lemniscatus* Released9 was estimated from a still video frame. As snake morphometrics are taken at the time of vouchering, SVL, mass, and sex were not directly measured for released individuals.

### Video analysis

We selected videos for analysis that included thrashing behavior with minimal translocation that stayed within the field of view of one camera (Fig. 1A). We used the Adobe Premiere Pro (Adobe Systems, San Jose, CA) built-in filter for GoPro Hero 4+ Wide angle to remove lens distortion. We wrote custom Matlab (Mathworks, Natick, MA) code to perform a projective transformation on each video frame, removing parallax distortions to produce an image for direct measurement.

**Fig. 2:**
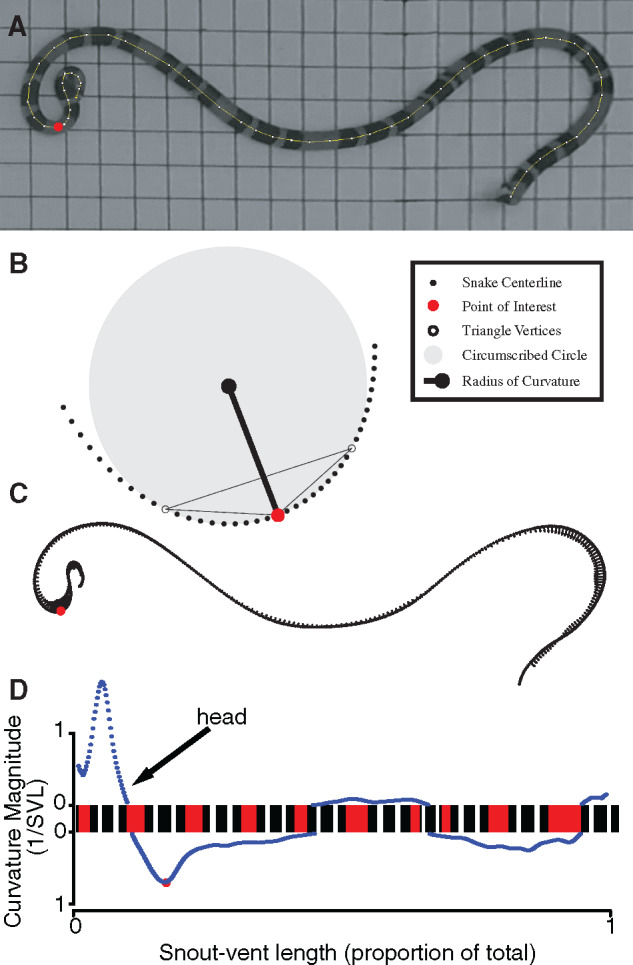
Methods for calculating the curvature of a snake. (**A**) Undistorted and rectified video frames at the conclusion of each bout of thrashing were traced by hand in ImageJ. (**B**) For each focal point in the snake centerline, the red focal point in B corresponds to the red focal point in B, the curvature was calculated as the distance from the point to a line connecting the 10th points on the left and right. (**C**) Curvature vectors are shown along each sampled point of the snake centerline as arrows. (**D**) The corresponding curvature magnitudes plotted to the right and left of a straightened depiction of a coral snake. The color pattern of the coral snake reflects the observed color pattern of this individual.

We used QuicktimePro 7 to watch the videos frame-by-frame and recorded the first and last frames that included motion as the start and end of each individual thrashing movement. Generally, a single thrashing bout was displayed in response to a single stimulus, and this pattern was repeated throughout the duration of the trial. We used ImageJ to measure the length of the snake in the video image, which was compared to measurements taken at the time of vouchering. We traced the centerline of the snake body at the end of each bout of thrashing with ImageJ (Fig. 2A).

We wrote custom Matlab code to analyze the centerline of the snake body. First, we resampled the traced centerline to produce 500 evenly spaced points (Fig. 2C). We corrected for noise in centerline tracing by smoothing the centerline using a moving average method with a span of 30 points. Each point along this smoothed centerline can be thought of as a point on the edge of a circle that provides the closest approximation to the body shape at that point. The angle of the tangent line of this circle may differ drastically from point to point in acutely curved areas. The curvature, which provides a measure of the difference in tangent line angle between neighboring points, is defined by the reciprocal of the circle’s radius.

We wrote code to automatically estimate the radius of curvature at each point along the body. The code first created a triangle using the point of interest and the 10th points to its left and right. Next, a circumscribed circle was constructed from the vertices of this triangle. The radius of curvature is the distance from the point of interest to the circumcenter of this circle (Fig. 2B). Because the lengths of these radii were measured in pixels, and because global curvature values correspond to different body configurations for different body sizes, each value was normalized by the total length of the snake centerline in pixels. The curvature was computed by taking the reciprocal. Reported here in units of 1/SVL, the curvature is a measurement of the tangent direction’s sensitivity among nearby points on a curve. Higher curvature values indicate more acute curves.

We did not compute the curvature for the anterior and posterior extrema with fewer than 10 points on a side (2% of body length). We used built-in Matlab functions to locate the areas of local maximum curvature along the body of the snake (e.g., the red dot in all panels of Fig. 2). If multiple local peaks were recorded in a 30-point window, the curvatures and their indices were averaged to 1 point.

To automatically determine whether a particular curvature vector was located to the right or left of a snake’s head, we wrote custom Matlab code to record the angle of each curvature vector in a snake-centerline coordinate frame. We defined the temporary snake-centerline axis as a vector pointing from the current point of interest toward its neighboring point, in the direction closer to the head of the snake. A curvature vector falling to the left of the snake-centerline axis indicated a curve to the left of the snake’s head (Fig. 2, red dot). With this method for computing curve direction, we calculated the percentage of left or right instances of local maximum curvature in each observation.

### Statistical approach

To maximize our inference ability and include information from all individuals, we first assessed whether snakes displayed any anti-predator behavior (e.g., thrashing, escaping, or head hiding) in every recorded trial of *Micrurus* behavior as a binary variable (presence or absence of a response). For the individuals that did respond, we then assessed the expression of that response using the number of thrashing events, their durations, and the body location and direction of the curvatures in each post-thrash pose. For the purpose of statistical modeling, we calculated the sum of the magnitudes of curvature for each of the 500 points along the body of the snake for each post-thrash pose, and we quantified each individual’s preference for a left or right head kink by determining the direction of the most anterior curve across multiple observations in the same trial. For each of these response variables, we constructed generalized linear mixed models (GLMMs) to test for effect of species, body size (SVL), sex, and stimulus type on the presence or expression of a response while accounting for individual collection ID as a random effect because every individual was tested more than once. For binary (Y/N) response tests of stimulus, we only included the treatments that had more than five observations per response category (contact, looming, and vibration), which removed 12 trials of 160 total. We also tested for the effect of collection site in the one species (*Micrurus lemniscatus*) that was collected from all localities, as there was otherwise high variability/stochasticity in which species was found at each collection site. If co-occurring species affect the anti-predator displays of individuals in a locality due to mimetic local adaptation, we would expect *M. lemniscatus* to have the highest behavioral variation because it was found sympatrically with every other species in our dataset. Finally, we tested for decay of the thrashing signal over the course of a trial by regressing thrash duration and sum of body curvature over time and assessing mean slope deviation from zero. All statistical models were built in R v 3.6.1 using the package “lme4” (Bates et al. 2015) for mixed modeling, and significance was assessed at α = 0.05 using number of groups in each GLMM as a conservative estimate of the degrees of freedom denominator.

To determine the thrashing frequency for each species, we divided the total number of observed thrashing events by the total amount of time each snake in the species was observed while encountering the experimental stimulus. The total amount of time excludes trials in which no thrashing was observed. Time in which the snake left the field of view was subtracted from this total time. To then compare these metrics across species, we bootstrapped data by randomly sampling with replacement from a combined dataset comprised of all individuals in a given species. To maintain the differences in propensity to thrash, these bootstrapped values were sampled in proportion to the thrashing frequency described above.

The color pattern of each snake was recorded from photographs captured at the time each snake was sacrificed to be vouchered as a museum specimen. A high-quality set of standard photographs was captured to record the color patterns. Then, the centerline of the snake was traced in ImageJ, and the locations of transitions between color bands were calculated based on **millimeter-**to-pixel ratio.

## Results

### Overall behavioral response

We recorded 160 behavioral trials in total across 25 snakes: 14 trials from 3 individuals of *M.* *annellatus*, 16 trials from 2 individuals of *M. hemprichii*, 71 trials from 12 individuals of *M. lemniscatus*, 30 trials from 4 individuals of *M. obscurus*, and 29 trials from 4 individuals of *M. surinamensis* (Table 1).

**Fig. 3:**
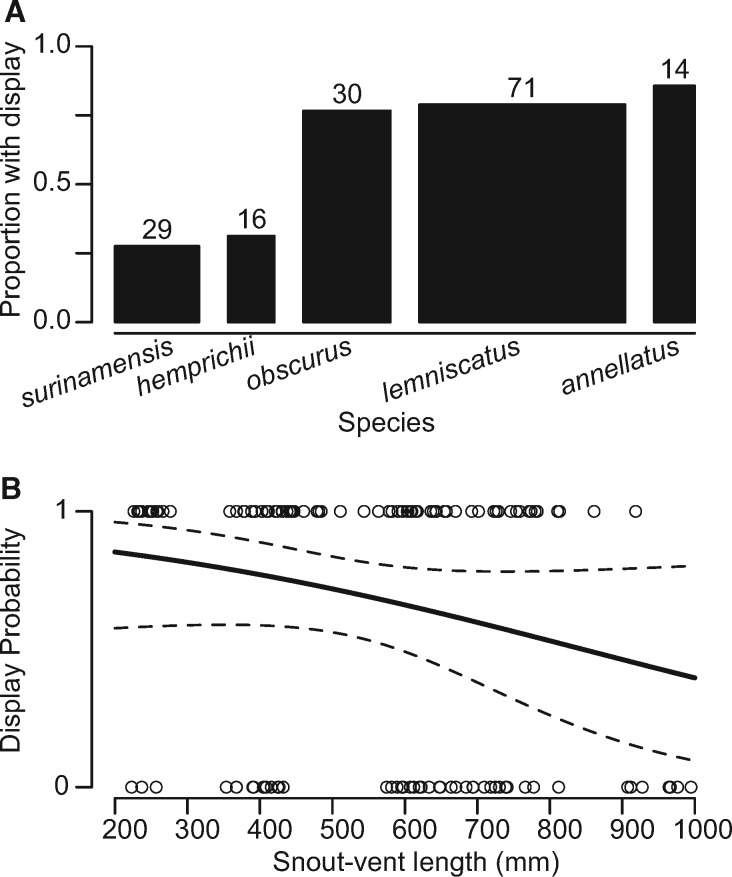
Not all individuals performed a thrashing display in response to all cues. Display probability depended most strongly upon (**A**) species identity (binomial GLMM: *F*_4,22_= 5.907, *P *=* *0.002) and (**B**) body size as measured by SVL (*F*_1,22_= 5.732, *P *=* *0.026). Fitted effect of SVL is predicted from a model that includes collection ID as a random factor, as all individuals were tested more than once. Width of bars and numbers above represent the number of individuals examined in each species.

Not all individuals responded to all predator cues in our trials. We found that the probability of response depended most strongly on an additive effect of both body size (binomial GLMM; *F*_1,22_= 5.732, *P *=* *0.026) and species (*F*_4,22_= 5.907, *P *= 0.002), with larger individuals and *M. surinamensis* and *M. hemprichii* least likely to respond (Fig. 3). We did not find a significant effect of collection locality (*M. lemniscatus* only, see “Methods” section; *F*_2,12_= 0.099, *P *=* *0.906) on the probability of response, nor significant interaction between SVL and species (*F*_4,17_= 0.665, *P *=* *0.625). We also found no effect of sex (*F*_1,22_= 0.780, *P *=* *0.387) or temperature (*F*_1,25_= 1.474, *P *=* *0.236) on display probability, although we note that most of our trials were conducted across a narrow range of body temperatures between 25.1°C and 27.8°C (20–80th quantiles). Although our ability to run some higher-order multiple regressions was limited by our sample sizes, the effect of stimulus type was marginally significant in a single fixed effect model (*F*_2,22_= 3.278, *P *= 0.057) and may interact with other effects. Observationally, response to a contact stimulus produced a response in every individual tested, but the probability of response varied across species and body sizes in looming and vibration trials.

When analyzing the thrashing display of the individuals that did respond to a predator cue, we found that thrash duration depended on body size (*F*_1,20_= 48.075, *P *< 0.001) and stimulus type (*F*_2,20_= 4.877, *P *= 0.019), with larger snakes and those presented with contact stimuli performing longer thrash displays. We also found that magnitude of body curvature in the post-thrash pose depended upon stimulus (*F*_2,20_= 5.601, *P *= 0.012) and species (*F*_4,20_* *=* *5.962, *P *=* *0.003), such that *M.* *lemniscatus* and those presented with a looming cue displayed the highest degree of body curvature (Supplementary Fig. S1). Sex had no effect on either magnitude (*F*_1,19_=* *0.212, *P *=* *0.651) or duration (*F*_1,19_=* *2.266, *P *=* *0.149; one unvouchered, unsexed individual excluded). We also found no effect of temperature on magnitude (*F*_1,20_= 0.284, *P *=* *0.600) or duration (*F*_1,20_= 0.155, *P *=* *0.697). We found no significant preference within individuals for directionality (or “handedness”) in local maximum curvature, as nearly all individuals turned both heads and bodies in both directions during display (mean proportion of local maximum body curves to the right: 0.495; mean proportion of poses with right-kinked heads: 0.430, but 17/20 individuals had proportions between 0.2 and 0.8). We also found no significant effect of body size, species, or stimulus on the number of thrashing events within trials (all *P *>* *0.05). Additionally, we saw no significant decay of the thrashing response over the course of trials, either in duration of thrash (mean slope: −0.003, range: −0.077 to 0.043) or degree of body curvature (mean slope: −0.288, range: −5.23 to 5.46). We did not have enough replicates to perform a robust test of locality effects.

### Intraspecific variation in *M. lemniscatus*

**Fig. 4:**
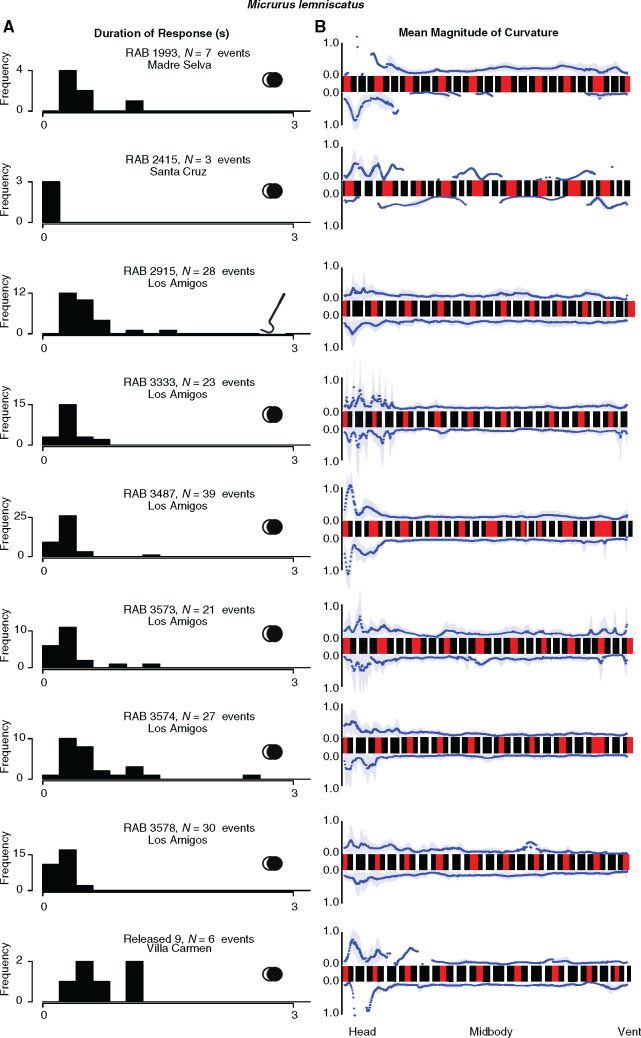
Intraspecific variation in thrash duration (**A**) and curvature (**B**) among individuals of *M. lemniscatus.* For each row of plots, the histogram of thrash durations on the left and the mean magnitude of curvature plot on the right represent the same trial. The stimulus used in each trial is depicted by the icon on the right side of the histogram: two overlapping circles denote a looming stimulus and a snake hook denotes a tactile stimulus. Color patterns of each snake reflect the observed color patterns of each individual measured to sub-millimeter resolution. Snake patterns are arranged such that the anterior portion is toward the left.

We compared the location and magnitude of body curvatures of the post-thrash pose among all individuals of each species. The variation among nine individuals of *M. lemniscatus* (Fig. 4) is emblematic of the intraspecific variation found in each of the other species as well (see Supplementary Figs S2–S5).

Overall, there is strikingly high consistency in thrash duration (Fig. 4A) and location and degree of body curvature (Fig. 4B) both within and among individuals of this species. Areas of highest curvature (highest peaks) are concentrated toward the anterior portion in each snake, demonstrating the stereotypical “neck kinking” mentioned in qualitative descriptions of this display. In most individuals, both sides of the body are used fairly equivalently in body curving (Fig. 4B). However, within the bounds of this stereotyped display, the ability of snakes to dynamically adjust display components across cues and ontogeny contributed to substantial intraspecific variation across all metrics. The longest duration of thrash occurred in RAB 3574 (Fig. 4A, seventh row), which was a generally active and moderately sized individual from Los Amigos. The most consistent and short durations of thrash were displayed by RAB 2415 from Santa Cruz (Fig. 4A, second row), which was generally inactive, and the smallest snake captured of this species (overall SVL effect on thrash duration also shown in Supplementary Fig. S1). As seen in the second row of Fig. 4B, this individual predominantly thrashed with the anterior portion of the body while keeping the rest of the body relatively stationary. There also appears to be substantial variation in how much of the anterior body displays the acute curves most associated with the neck, as well as how much of the posterior body displays the acute curves associated with tail coiling (not analyzed here; see in particular RAB 3573 and 3333). Overall, there appears to be a similar amount of variation in behavior as there is in coloration (precise color variation shown in Fig. 4B, see figure caption), even though both traits are expected to be under exceptionally strong stabilizing selection.

### Interspecific variation

**Fig. 5:**
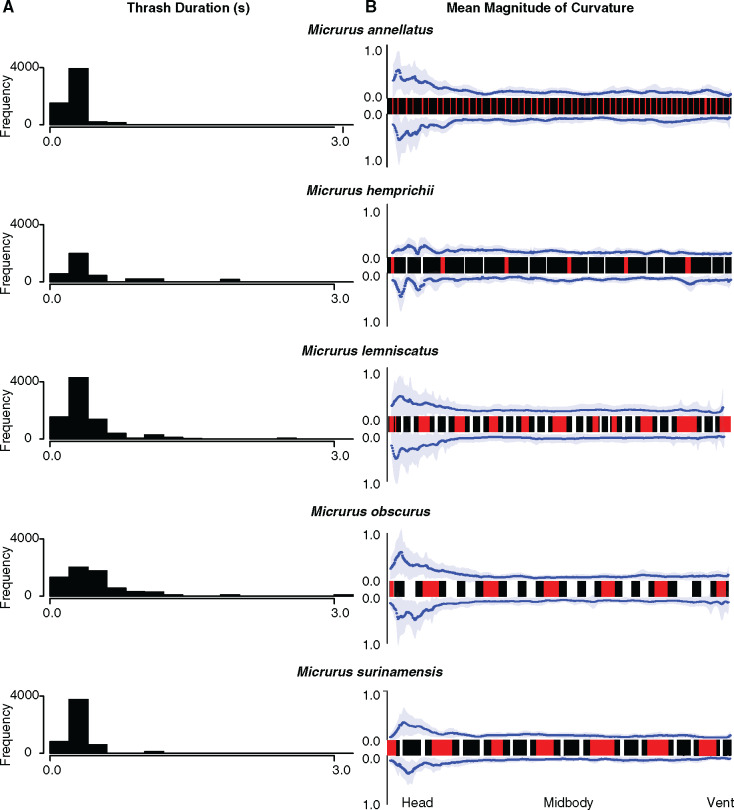
Interspecific variation in thrash duration (**A**) and curvature (**B**). Thrash durations for each species on the left are sampled with replacement proportionally by the ratio of events to total observed frames of video and plotted as histograms. On the right, the mean magnitude of curvature at each point along the body is plotted for one trial for each species in response to looming stimuli, with the exception of *M. surinamensis*, which responded to a vibratory stimulus. Color patterns of each snake reflect the observed color patterns of each individual measured to sub-millimeter resolution. Snake patterns are arranged such that the left side is anterior.

We found a similarly high consistency across species in the major features of the thrash display: the duration of thrashing tended to be relatively short (median value below 0.5 s for all species; Fig. 5A) and all species tended to have the largest magnitude of curvature toward the anterior portion of the body, irrespective of substantial variation in typical coloration and patterning (Fig. 5B; see also Supplementary Figs S2–S5). Beyond these major features, however, we again found variation in multiple components. The difference between the mean curvature at midbody and the mean curvature at the head was highest in *M. lemniscatus* and *M. obscurus*, and lowest in *M. hemprichii* and *M. surinamensis*. Similarly, the region of higher curvature extended posteriorly from the head much further in *M. lemniscatus* and *M. obscurus* than in *M. hemprichii*, *M. surinamensis*, and *M. annellatus*. However, posterior body curving near the tail was highest in *M. obscurus*, which happens to be the species with the shortest relative tail length in our dataset. This pattern may indicate compensation for a shorter tail by involving more of the body in the thrashing display. Additionally, the variance in thrashing durations was surprising, as longer bouts of thrashing were not equally likely for all species. *Micrurus annellatus* was the least likely to display for more than 1 s, *M. surinamensis* and *M. hemprichii* always displayed for less than 2 s, while *M. lemniscatus* and *M. obscurus* thrashing lasted up to 3 s (Fig. 5B).

## Discussion

By analyzing the behavior of non-locomotory coral snake anti-predator behaviors, we provide a new approach for quantitative comparison of critical signaling elements both within and among species. These results have important implications for testing theoretical expectations of mimicry systems and comparing results to other mimicry systems, such as butterflies, in which all players are chemically defended. Following theoretical predictions of these Müllerian systems, we expected to find the greater behavioral similarity among sympatric species than intraspecific variation across multiple sites or even just high similarity across all individuals irrespective of species or locality due to their high intraspecific similarity in color pattern (Fig. 4; Supplemenatry Figs S2–S5). Contrary to this expectation, we found surprising diversity in (1) propensity to display at all, (2) duration of thrashing, and (3) distribution of body curvatures during the display, with no clear relationship to patterns of species sympatry. For context, if this level of variation had been present within color phenotype, it would be analogous to some individuals having no warning colors at all (e.g., unmarked brown coloration) despite their toxicity. In particular, we found that this signal can be dynamically adjusted across ecological contexts, such that small snakes and those under physical contact by a predator are the most likely to respond and produce the most vigorous responses.

### Ecologically relevant variation in signal construction: what matters?

Our superficially paradoxical results add to mounting evidence that the drivers of trait variation in mimicry systems are not well understood (Joron and Mallet 1998; Mallet and Joron 1999; Cox and Davis Rabosky 2013; Davis Rabosky et al. 2016a). Although one interpretation could be that South American coral snakes are not part of a mimicry system, a more likely possibility is that the lethal levels of toxicity allow for greater trait variation due to the higher cost to predators that erroneously identify prey (Pough 1988). In particular, our results suggest that some aspects of the behavioral display—a short thrash duration and an acutely kinked neck—may be more important for effective signaling to predators than other traits, such as which direction the body curves or the degree of curvature beyond the neck. Although variation in color pattern has been well documented and is considered the most important trait for communicating the potential for chemical defense, the variation we observe in *Micrurus* displays specifically in response to experimental cue supports the idea that behavior also plays a key role in deterring predation. More broadly, our ability to use a single method to analyze anti-predator display across a clade with a divergence date of at least 10 million years (Pyron et al. 2013) gives some appreciation for how long the main thrashing element of this display has been maintained within the coral snake lineage. However, our results suggest that simplistic models of signal canalization in mimicry systems may benefit from expansion that accounts for the ways in which signals can and do still vary within and among species.

### Challenges of studying non-locomotory behavior in venomous coral snakes

The non-locomotory movements of snakes present several unique challenges to quantitative biomechanical analyses. To enable translocation of the snake body toward a distant goal, many forms of snake locomotion involve either linear or roughly sinusoidal body shapes to maximize contact between the snake body and the substrate or act as a continuous airfoil (Lissmann 1950; Jayne 1986; Moon and Gans 1998; Hu et al. 2009; Socha 2011; Titcomb et al. 2014). Self-occlusion necessarily reduces the effective surface for locomotion. Non-locomotory behaviors are less mechanically constrained by the requirement to have forward substrate-reaction forces sum to a positive value and therefore can include self-occlusion; balling, coiling, rolling onto the dorsal surface are common components of anti-predator behaviors that are rarely observed during locomotion (see Davis Rabosky et al. 2019). The extremely elongate body form and high degrees of freedom conferred by the highly articulated musculoskeletal system result in more acute curves than observed in other model organisms with elongate body forms, especially those that lack a complex musculoskeletal system (e.g., *Caenorhabditis elegans*, Padmanabhan et al. 2012; Brown et al. 2013a). Such extreme self-occlusions make it difficult to use automated tools for passively tracing the snake centerline.

Furthermore, the banded color patterns and self-mimicry by the tail of the head (Greene 1973) make it difficult to identify the morphological features of a snake from an isolated video frame without context. These features likely contribute to snake fitness by making it difficult for a predator to target an attack toward the head (Brodie 1993) and have resulted in a particularly challenging dataset for existing computer vision tools.

While visual fiduciary markers placed on the snake can aid in collecting precise kinematic data (e.g., Gart et al. 2019), the handling required to affix such markers likely alters the behavioral response of the snake. Furthermore, the highly toxic venom of these snakes, together with impressively adhesive-resistant skin lipids (Torri et al. 2014), makes it impractical and unsafe to attempt such marking with wild-caught venomous snakes during a herpetological survey in relatively remote locations with limited infrastructure.

The smooth surface of the arena, selected to ensure thorough cleansing, resulted in lower substrate friction for the snake. Although some slipping was observed during thrashing, recent studies suggest that snake motor control is independent of surface friction (Schiebel et al. 2019). From our observations, the snakes were able to precisely control the mechanics of their anti-predator displays, including rapidly inducing and halting movement, maintaining elevated postures, and tail coiling. The stationary periods between thrashing bouts did not include any slow sliding or slipping, which would indicate that the snake’s behavior is dependent on friction with the substrate. Encountering an artificial substrate may induce alterations of the motor signals that control the behavior. However, the artificial substrate was applied to all trials, so the relationships among the species should be maintained, even if there is an effect of substrate. Thus, we argue that our results likely reflect snake behavioral variation on more natural substrates.

### Future directions

We demonstrate that a video-based approach can be feasibly applied to quantitatively examine the non-locomotory behaviors of snakes under semi-natural conditions. This approach facilitates ecologically relevant biomechanical inquiry with strong evolutionary impact. While we recognize that our stimuli, especially the vibratory stimulus, may not be a perfect match to those provided by potential predators, at least one individual responded to every category of stimulus with an anti-predator display.

In addition to venomous coral snakes, distantly related snakes with varying toxicity have independently converged several times on these conspicuous color patterns and thrashing displays (Greene 1979; Davis Rabosky et al. 2016b). Just as previous studies have leveraged a quantitative analysis of color pattern to measure convergence and experimentally examine the effect on predation rates (Brodie 1993; Buasso et al. 2006), the analysis presented above enables more precise measurement of convergence and an experimental approach for determining the effect of behavior on predation rates. Since snakes of all sizes, and consequently all ages, display vigorous thrashing behavior in the absence of any parental care (Shine 1988), this anti-predator response has likely evolved as an innate response, with little learning over the course of a lifetime. In this case, quantitative characterization of anti-predator behaviors can be modeled as phenotypic traits to gain insight into the evolutionary processes underlying the patterns of behavior observed in nature.

## Supplementary Material

obaa006_Supplementary_Data

## Data accessibility

Videos are archived and freely available at https://doi.org/10.7302/bksb-t580. Code to compute the curvature of the snakes is posted at https://github.com/sdanfort/snake_curvature.
